# Towards the scale-up of the formation of nanoparticles on α-Ag_2_WO_4_ with bactericidal properties by femtosecond laser irradiation

**DOI:** 10.1038/s41598-018-19270-9

**Published:** 2018-01-30

**Authors:** Marcelo Assis, Eloisa Cordoncillo, Rafael Torres-Mendieta, Héctor Beltrán-Mir, Gladys Mínguez-Vega, Regiane Oliveira, Edson R. Leite, Camila C. Foggi, Carlos E. Vergani, Elson Longo, Juan Andrés

**Affiliations:** 1 CDMF-UFSCar, Universidade Federal de São Carlos, P.O. Box 676, CEP, 13565-905 São Carlos, SP Brazil; 2 Department of Inorganic and Organic Chemistry, University Jaume I (UJI), Castelló, 12071 Spain; 3 Institute for Nanomaterials, Advanced Technologies and Innovation Technical University of Liberec, Studentská 1402/2, 461 17 Liberec, Czech Republic; 4 GROC∙UJI, Institut de Noves Tecnologies de la Imatge (INIT, University Jaume I (UJI), Castelló, 12071 Spain; 5 FOAr-UNESP, Universidade Estadual Paulista, P.O. Box 1680, 14801903 Araraquara, SP Brazil; 6 Department of Analytical and Physical Chemistry, University Jaume I (UJI), Castelló, 12071 Spain

## Abstract

In recent years, complex nanocomposites formed by Ag nanoparticles coupled to an α-Ag_2_WO_4_ semiconductor network have emerged as promising bactericides, where the semiconductor attracts bacterial agents and Ag nanoparticles neutralize them. However, the production rate of such materials has been limited to transmission electron microscope processing, making it difficult to cross the barrier from basic research to real applications. The interaction between pulsed laser radiation and α-Ag_2_WO_4_ has revealed a new processing alternative to scale up the production of the nanocomposite resulting in a 32-fold improvement of bactericidal performance, and at the same time obtaining a new class of spherical Ag_x_W_y_O_z_ nanoparticles.

## Introduction

The ever-increasing use of semiconductors in daily human life has brought a wave of new materials with a wide range of technological applications. In particular, one of the semiconductor families that has attracted the most attention in recent years is the family of ternary tungsten oxides, such as metal tungstates (with the general formula MWO_4_), which are a class of functional materials with fascinating properties at the cutting edge of fundamental science, as well as having many potential applications that are studied and applied widely in many fields^1–3^.

Within this family of materials, silver tungstate (α-Ag_2_WO_4_) is an important inorganic material that has attracted significant attention owing to its applications in photocalalysts, photoswitches, or as an alternative to conventional wide band gap semiconductors^4–13^. Recent studies have demonstrated that crystals of α-Ag_2_WO_4_ showed a metal nanoparticle growth attached to the semiconductor framework, which in the near future may lead to outstanding applications due to Ag segregation, such as photocatalysis, ozone sensing and bacteriocidicity^3,5,8,14–22^.

Despite the great potential for growing novel metal nanoparticles on the framework of tungstate semiconductors, the ability to grow and control their molecular structure has been limited to Transmission Electron Microscopy (TEM) processing^2,23–31^. It is of exceptional scientific importance to scale up the production of the assembly under non-special conditions, and at the same time the methodology should provide enough versatility to be extensible to a wide range of materials. In this context, ultrashort laser irradiation over the sample in air may represent an alternative, as it can be easily integrated within a production process. Other alternatives may be electron beam machining.

The influence of laser irradiation over materials promotes a complex interplay between various physical and chemical mechanisms associated with sub-nanosecond timescale processes, influencing the constituent elements of materials in an intense way, which may result in the emergence of changes in the macroscopic behavior of materials due to internal modifications. In this sense, the current scientific literature demonstrates an ever-increasing use of femtosecond laser radiation in processing materials, the induction of a nanometric structure in bulk materials being its most popular use for obtaining novel species that present highly attractive properties for technological advances^32–37^. However, the ease with which the structure of materials can be modified at nanometric scales also allows the manufacture of novel complex nanoensembles^38–41^. Nowadays, the femtosecond laser-matter interactions that lead to the production of such promising materials are still not fully understood. However, the reliability and versatility of using pulsed laser radiation for nanomaterials has led to an exponential growth in its use^42–44^.

Based on this fact, in this communication we seek to fulfill a three-fold objective. The first is to report the novel formation of Ag and (Ag_x_W_y_O_z_) nanoparticles from α-Ag_2_WO_4_ by laser-assisted irradiation, where a large amount of material is created and non-special conditions are required, rather than irradiating the α-Ag_2_WO_4_ sample in air. These results are compared with those obtained in previous theoretical and experimental studies on the response triggered by electron beam irradiation on α-Ag_2_WO_4_. Second, based on this comparison, an explanation is given to unveil the formation of Ag nanoparticles occurring along the laser interaction. The third aim is to demonstrate that laser-irradiated samples have a much higher bactericidal activity than samples treated with electron beams. We believe that these novel results are of outstanding relevance, since they may inspire the efficient synthesis of nanocompounds and contribute to broadening the fundamental knowledge on the effect of laser-matter interactions.

## Experimental Results

In this proof-of-concept experiment to demonstrate the ability of the femtosecond laser to increase the efficiency of the production of nanoparticles over large areas of α-Ag_2_WO_4_ and test its bactericidal activity, an 8 mm × 8 mm portion of the surface of each sample was irradiated. The samples were then characterized by high resolution TEM (HR-TEM) and analyzed by energy dispersive X-ray spectroscopy (EDS). In parallel, bactericidal activity was tested by bringing the treated samples into contact with Planktonic cultures of Methicillin-Resistant Staphylococcus Aureus ATCC 33591 (MRSA), and then analyzing them by scanning microscopy (SEM) and confocal laser scanning microscopy (CLSM).

### Material characterization

Powder and pellet samples of α-Ag_2_WO_4_ were prepared and irradiated following two different routes. Protocol I consisted in irradiating the sample with a spot diameter in the order of 20 µm at a fluence of about 60 J/cm^2^. In protocol II the sample was irradiated with a spot diameter of approximately 84 µm at a fluence of about 3.6 J/cm^2^. The samples were then characterized by HR-TEM and analyzed by EDS.

**Figure 1:**
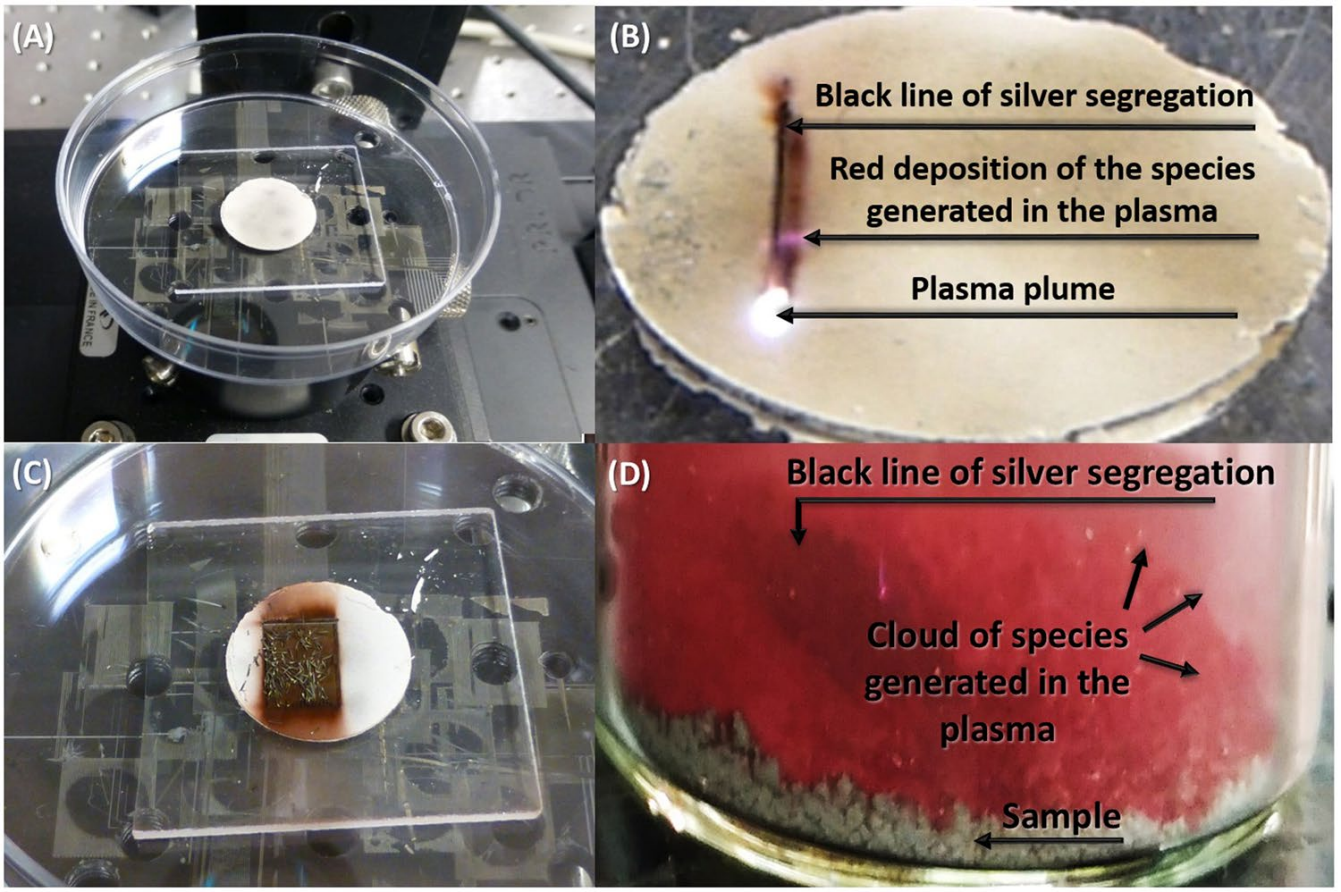
Photographs of sample (**A**) before, (**B**) during, and (**C**) after the laser irradiation process. (**D**) Detail of the cloud of species generated in the plasma.

In protocol I, two different chemical regions can be distinguished based on the change in color of the material. On the one hand, the laser interaction leaves a clear black line on the sample whereas, on the other hand, the extremely high density of photons also leads to the formation of a plasma plume which produces a red cloud that can deposit with time, as can be observed in Fig. 1, where the samples are exhibited under the laser irradiation process.

**Figure 2:**
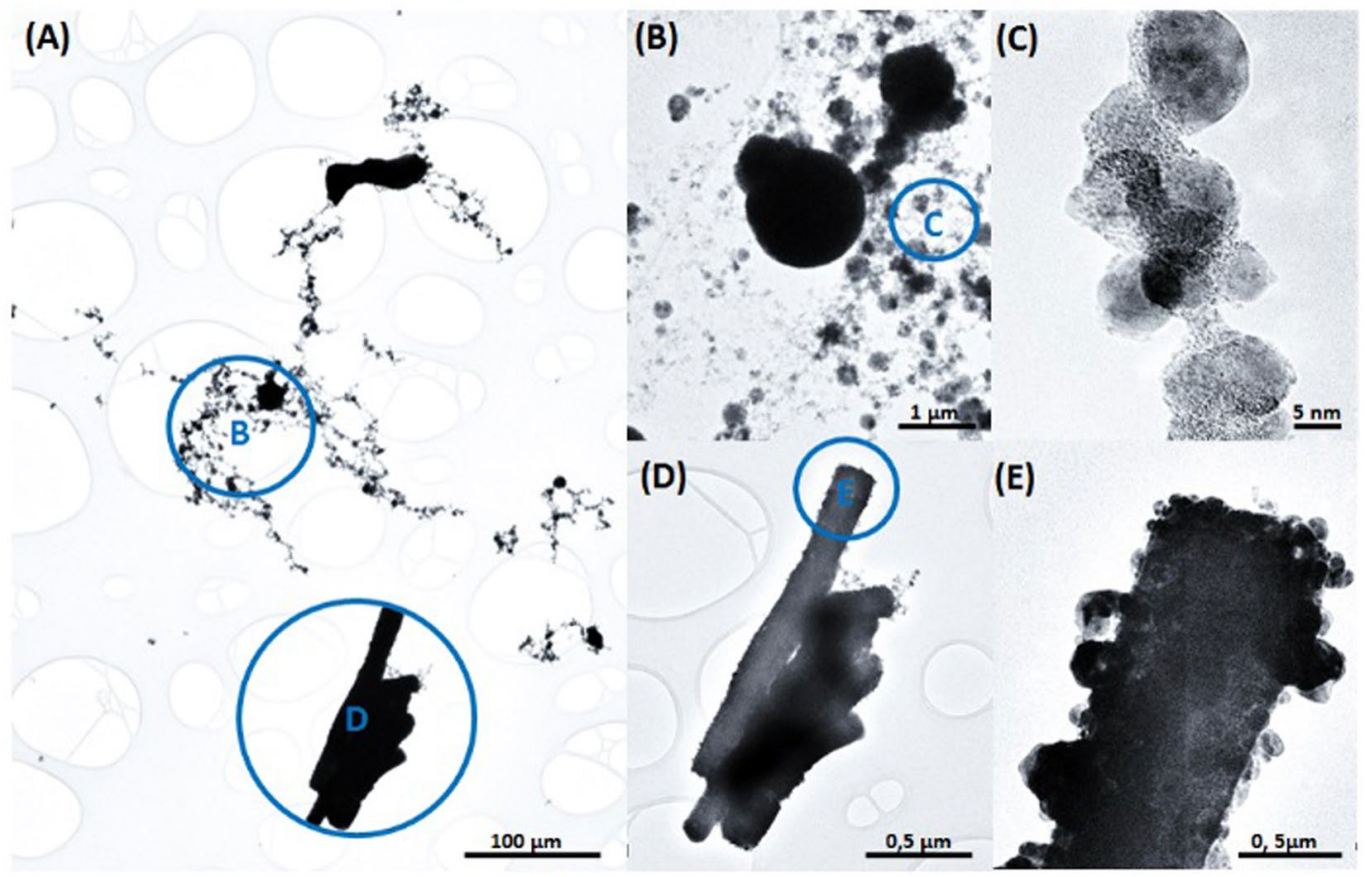
(**A**) Sample irradiated with a femtosecond pulsed laser beam (black part of the sample); (**B**–**C**) Magnification of spherical nanoparticles formed by laser; (**D**–**E**) Magnification of nanorods containing nanoparticles on the surface.

Figure 2A depicts a low-magnification HR-TEM image of the regions obtained from the black line acquired in protocol I. Two different types of microstructures can be observed: spherical nanoparticles (Fig. 2B and C), which can be isolated or agglomerated, and nanorods sputtered from the sample (Fig. 2D). Additionally, in Fig. 2E it can be seen that there was a release of nanoparticles in specific regions of the α-Ag_2_WO_4_ nanorod.

**Figure 3:**
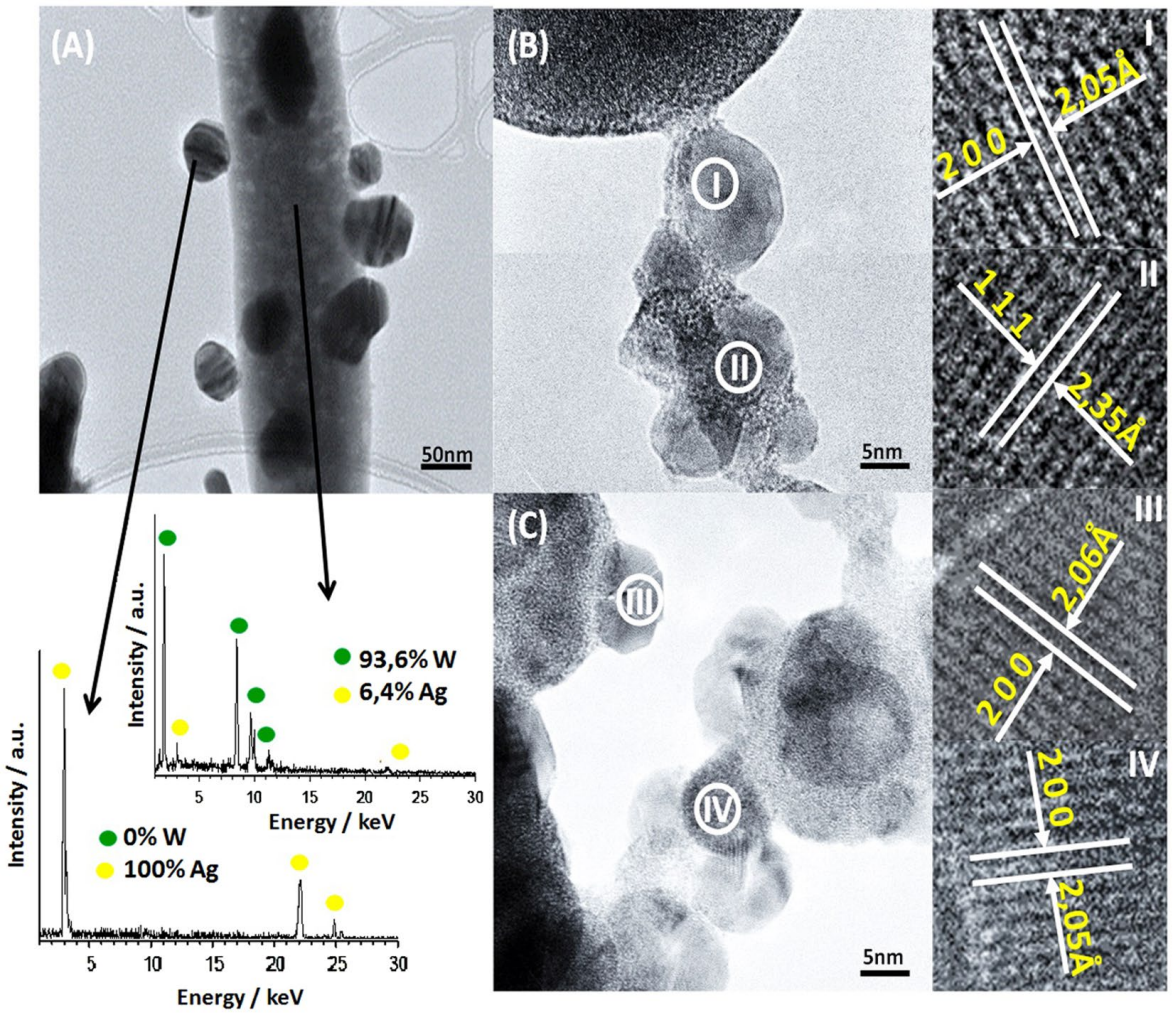
(**A**) HR-TEM image of the α-Ag_2_WO_4_ sample irradiated by laser and EDS analysis of rods and NPs; (**B**) and (**C**) detail of the Ag NPs formed; I, II, III and IV crystallographic planes of Ag NPs.

EDS analysis was performed on the irradiated sample (Fig. 3). In Fig. 3A, spherical nanoparticles are observed on the surface of a nanorod. EDS analysis of these nanospheres shows only the presence of Ag (100 at % Ag), whereas Ag and W (atomic percentage ratio Ag/W = 0.068) are observed in the center of the nanorod. The last atomic percentage ratio, less than 2, reveals that there has been a reduction of Ag^+^ to Ag^0^ and, thus, metallic silver has been segregated on the tungstate surface. Therefore, the formula of the nanorod becomes α-Ag_2-x_WO_4-δ_.

As can be observed in Fig. 3B and C, most of the spherical nanoparticles are agglomerated and they exhibit a crystalline structure. The surface of these spherical nanoparticles is very smooth, with sharp clear edges, which indicates a single-crystalline nature. This is confirmed by the reciprocal distances with the stronger reflections corresponding to lattice planes (200) and (111), which matches cubic Ag, according to the JCPDS database (PDF 65-287). These results suggest a strong decomposition of α-Ag_2_WO_4_ and the reduction of Ag^+^ to Ag^0^ under laser irradiation.

**Figure 4:**
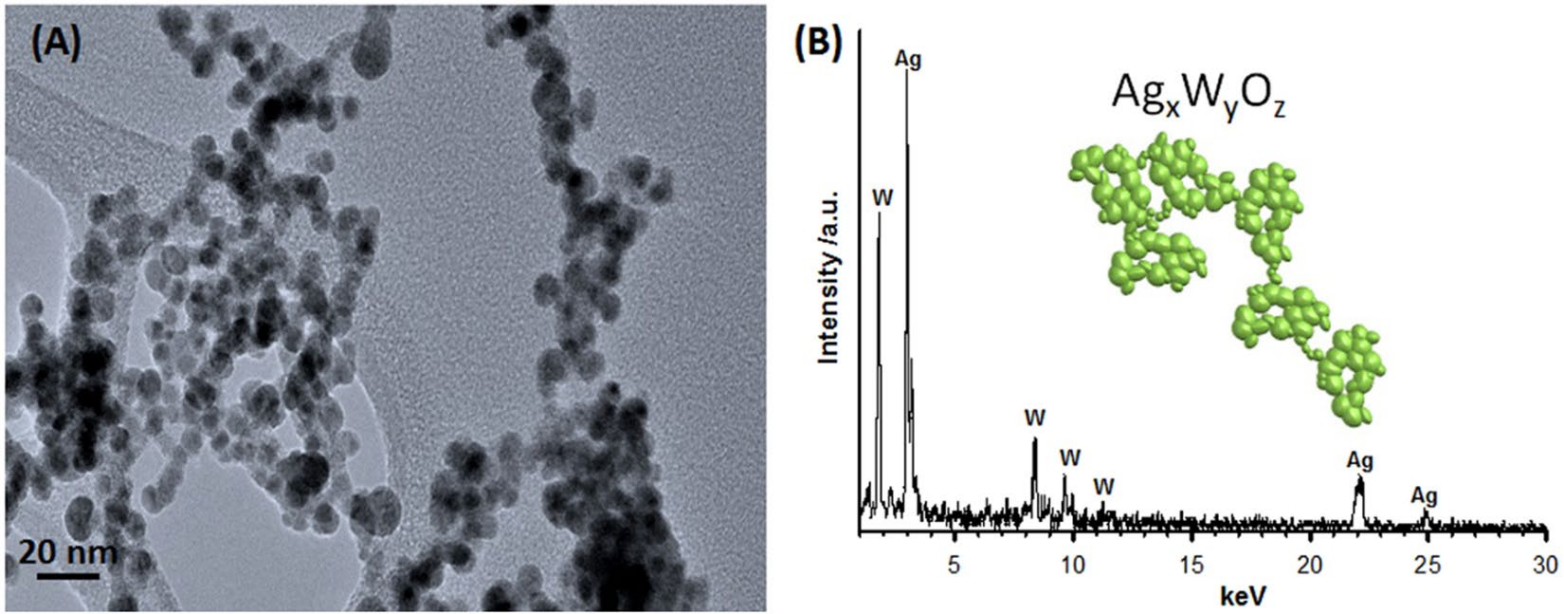
(**A**) Higher magnification HR-TEM micrographs of the particles contained in the plasma (red region); (**B**) EDS analysis of these particles (Ag_x_W_y_O_z_). Note that the oxygen signal is not included in the figure.

Next, it is important to focus attention on the species that form the red cloud produced by the plasma plume, the species that effectively leave the material’s surface. To analyze these species, we collect them directly from the red cloud. The morphology of the collected material is shown in Fig. 4A and a compositional analysis of single particles by EDS is indicated in Fig. 4B. All the particles that form the red cloud are spherical and with a particle size of less than 10 nm. They contain Ag, W and O with different stoichiometry, the formula for which can be denoted as Ag_x_W_y_O_z_ due to the non-homogeneity of this material.

In protocol II, at a lower irradiation fluence regimen, there is no observable ablation plume and only a clear black line in the irradiation zone can be distinguished. The species found here don’t show any appreciable differences from the species coming from the black line in protocol I. Consequently, when only the formation of Ag nanoparticles on α-Ag_2_WO_4_ is desired, low energies or high irradiation speeds can be employed and Ag segregation can be optimized.

**Figure 5:**
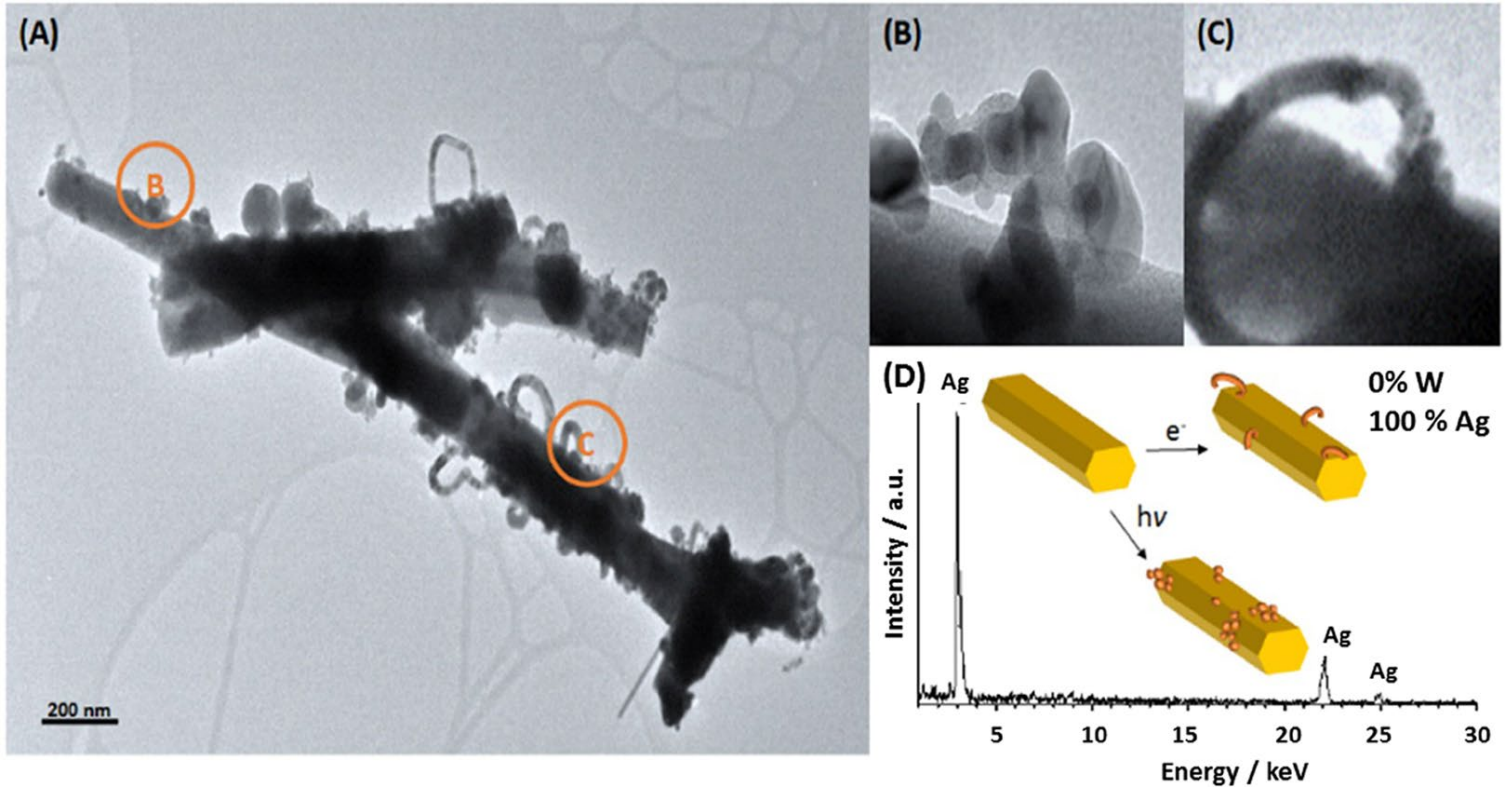
(**A**) HR-TEM image of the α-Ag_2_WO_4_ sample irradiated by laser followed by subjecting it to irradiation with an electron beam; (**B**) Ag NPs formed by laser irradiation; (**C**) Ag NPs formed by electron beam irradiation; (**D**) EDS analysis results of spherical Ag NPs and the Ag filaments which show the scheme for obtaining the different Ag NPs.

Next, it is important to compare the results obtained when α-Ag_2_WO_4_ crystals were irradiated by laser with those observed when these samples were irradiated with an electron beam in the TEM under a high vacuum^17,26^. In the latter, the formation of Ag filaments on the surface of α-Ag_2_WO_4_ is identified, and the crystalline structure of α-Ag_2_WO_4_ remains unchanged. So there are differences between these two processes. To clarify them, Fig. 5A shows an α-Ag_2_WO_4_ sample irradiated by laser followed by subjecting it to irradiation with an electron beam for a short time (5 min average). The Ag produced by laser irradiation presents a spherical morphology on the surface of the semiconductor and these spheres do not grow with the time of electron exposure (Fig. 5B). Conversely, in the regions of the sample without previous Ag segregation, the formation of Ag filaments on the surface is observed, due to the irradiation by the electron beam (Fig. 5C). EDS confirmed that both nanostructures contain only metallic Ag (see Fig. 5D). To see the growth of the filament in detail, the reader is directed to Fig. S1 of Supplementary Material.

The complete mechanism of the interaction between short pulsed laser radiation and matter is rather complicated. The energy of the laser pulse is absorbed by the electrons on the sample’s surface, which is a practically instantaneous process. The consequently excitation of electrons contrast with the electrons in the lattice that are in a basal state, and this excitation gradient leads to an equilibrium that is reached through rapid electron-phonon interactions as well as electron diffusion out of the excited region. At the point when the lattice excitation is high enough, ablation may occur. In the case of the electron irradiation, the Ag nanoparticles grow in sequential steps, suffering changes in size and shape along irradiation, due to the presence of electrons on conduction band. This leads to a particle segregation process that is associated to a kinetic energy exchange.

In sum, when we irradiate the sample first with the laser and later with the electron beam, the main information that we can extract is the following: i) both mechanisms allow the segregation of Ag, ii) the nanoparticles obtained with the laser are spherical, while the segregation of Ag in the electron beam promotes the formation of filaments, and iii) in the places where the nanoparticles have already been segregated by the laser, there is no longer any segregation by the electron beam.

X-ray diffraction (XRD) was also performed to analyze the differences between femtosecond laser irradiated material and non-laser irradiated material (Fig. S2 in the Supplementary Material). A small displacement of the peaks in the X-ray pattern of the laser irradiated material occurs, which indicates that there has been a shift in the distance between the planes of the lattice, thereby distorting the original structure without any extra peaks appearing. The Ag NPs formed by the process cannot be observed using XRD in the powder technique, although it is evidenced in the HR-TEM measurements.

### Bactericidal Behavior

A comparison of bactericidal activity against MRSA was performed between non-treated samples of α-Ag_2_WO_4_, and α-Ag_2_WO_4_ treated with laser radiation.

**Figure 6:**
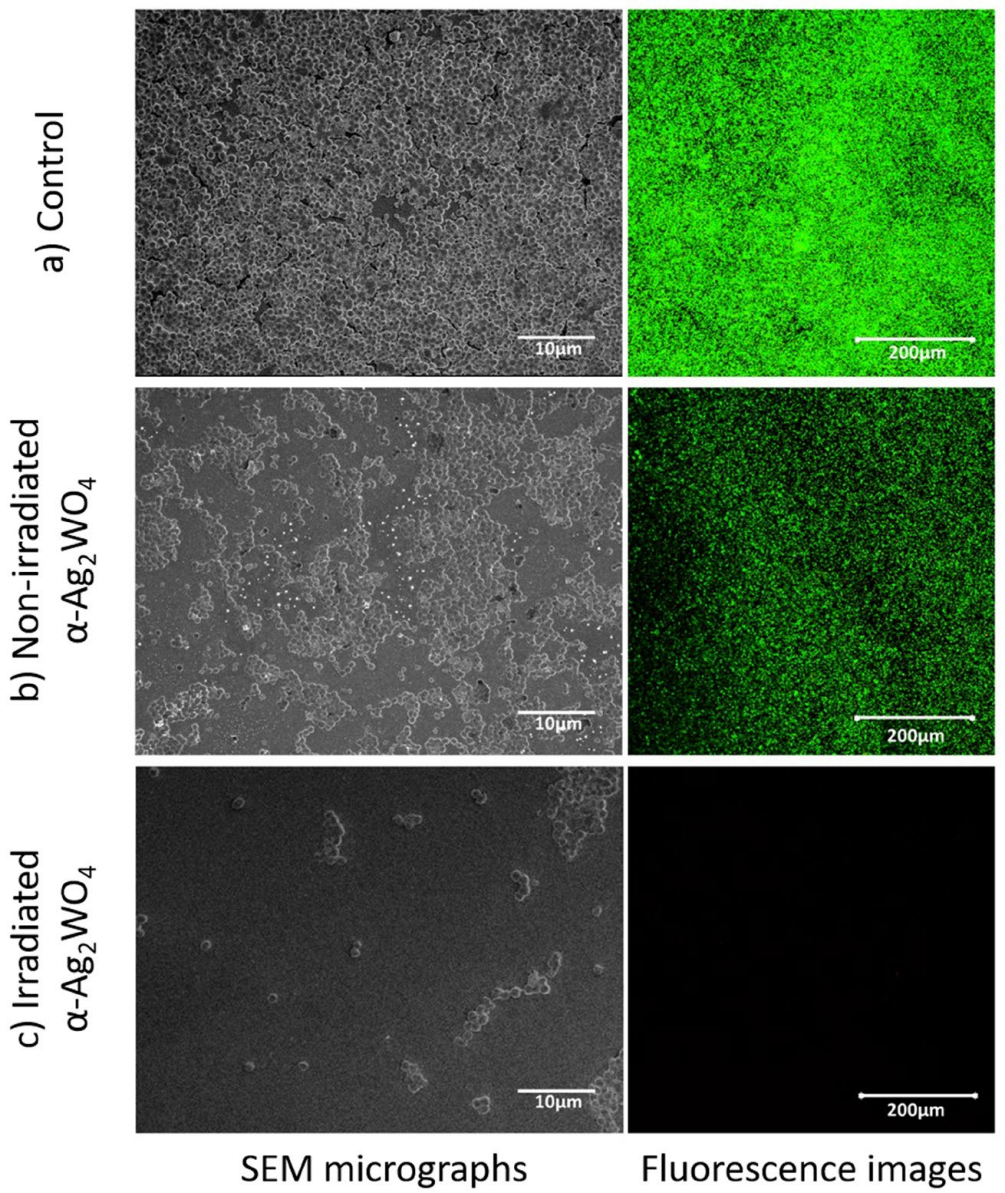
SEM micrographs and fluorescence images of (**a**) pure planktonic culture of MRSA, (**b**) MRSA culture after coming into contact with 125 µg/mL of non-treated α-Ag_2_WO_4_, and (**c**) MRSA culture after coming into contact with 3.91 µg/mL of α-Ag_2_WO_4_ irradiated with laser radiation.

The bactericidal activity of materials should lead to complete inhibition of the growth of a particular bacteria or fungus. The minimal inhibitory concentration (MIC) of the samples was measured by exposing planktonic MRSA cells, previously incubated in a 96-well microtiter plate for 24 hrs at 37 °C, to a series of dilutions of α-Ag_2_WO_4_ non-treated and laser-treated samples in tryptic soy broth (TSB)(from 1000 to 1.95 µg/mL). Pure TSB mixed with MRSA was used as a control, and the lowest concentration of α-Ag_2_WO_4_ where there was no visible bacteria growth was considered as the MIC. Figure 6 shows a difference in the bacteria growth colony after coming into contact with non-irradiated and irradiated α-Ag_2_WO_4_ samples. The results demonstrate that, while the non-irradiated material shows a MIC at 125 µg/mL, when the material is irradiated with laser radiation, there is a 32-fold improvement in MIC for 3.91 µg/mL over non-treated samples. Moreover, studies performed by irradiating with an electron beam report a MIC magnification of only about 4 times for 31 µg/mL^45^.

The literature indicates that the improvement is due to the formation of Ag NPs on the surface of α-Ag_2_WO_4_, which, in accordance with several studies, present an excellent bactericidal activity against various microorganisms^46–49^. The difference between electron beam-treated samples and laser radiation-treated samples may be due to the amount and/or the morphology of Ag segregated by each treatment. While electron beam treatment is limited to TEM processing, growing Ag with a non-specific morphology, laser irradiation treatment allows the segregation of spherical NPs of Ag in large areas of α-Ag_2_WO_4_.

## Discussion

**Figure 7:**
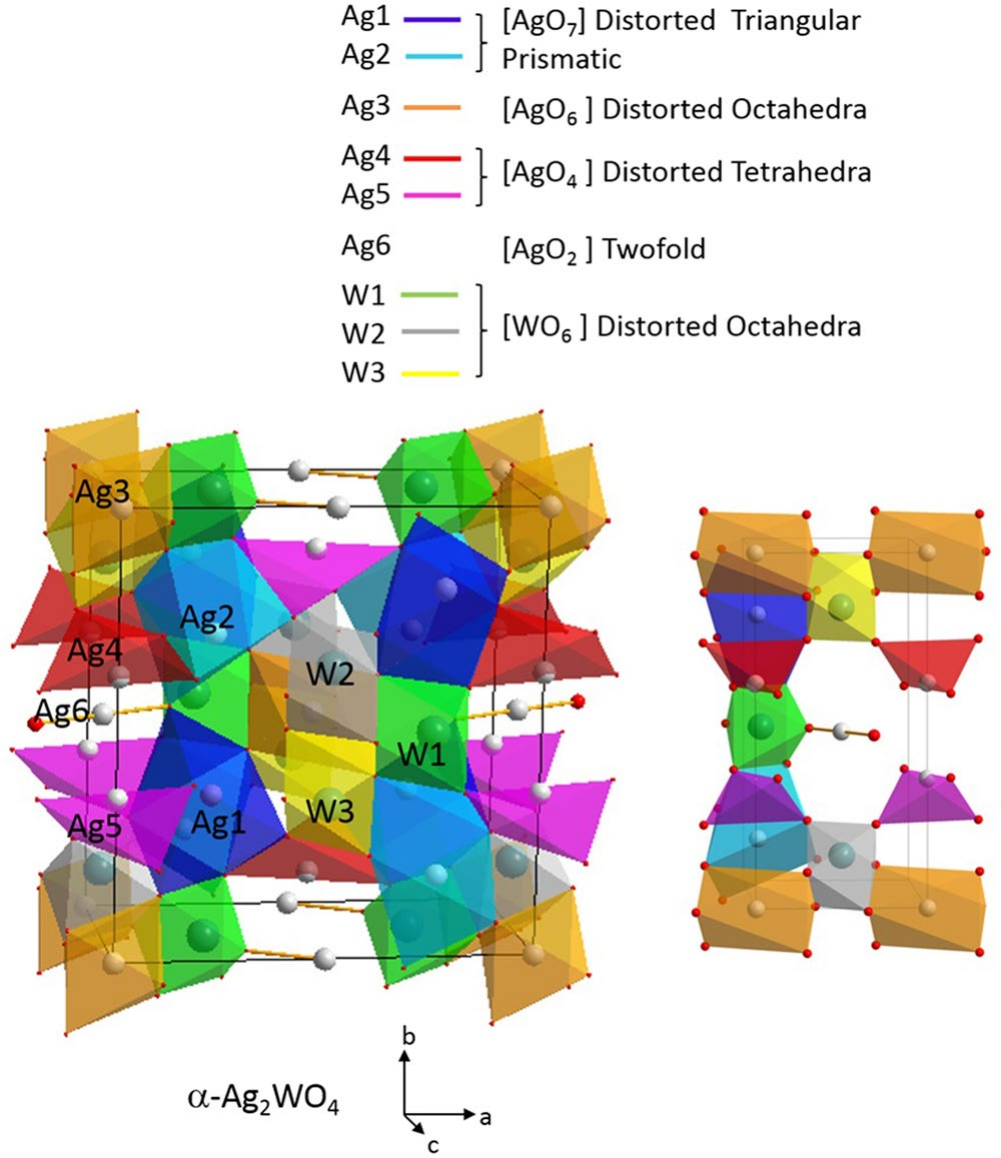
α-Ag_2_WO_4_ crystal structure.

To gain a better understanding of the resultant species it is important to associate their growth to energy-dependent effects. α-Ag_2_WO_4_ is a semiconductor with a band gap of 3.1 eV, i.e., 400 nm, which cannot be activated by visible light^50^. From detailed experimental and theoretical studies it is well established that their orthorhombic structure can be described by the local coordination of both W and Ag cations, i.e., ([AgO_y_] _y_ = 2, 4, 6, and 7): angular [AgO_2_], tetrahedral [AgO_4_], octahedral [AgO_6_], pentagonal bipyramid [AgO_7_], and [WO_6_] clusters^2,23–25^. These clusters are intrinsically disordered, and the Ag-O and W-O bonds, as well as the O-Ag-O and O-W-O bond angles, are free to stretch/shorten and bend, respectively, as shown in Fig. 7. This could affect the crystal field and change the dipole and electronic band structures of both the valence band (VB) and the conduction band (CB), thereby influencing the behaviors of photogenerated charge carriers, including the excitation processes^2,14,21^.

When the sample is exposed to sub-picosecond pulsed laser irradiation, the laser radiation is delivered to the surface of the material finding it in a stationary state, which means that there are no interatomic movements and external electrons of atoms in the surface of the material can absorb incoming photons, thereby yielding a photo-activation process. The excess of energy in the electrons would lead to an energetic jump to a metastable excited state, and considering that in sub-picosecond laser pulses a large population of photons is interacting with the excited electrons in a shorter time than that required by electrons to return to the basal state, one of the possibilities that can occur is that electrons experience multiphoton absorption, leading to the expulsion of electrons out of the atom’s structure and the laser-matter interaction would be limited to these effects or further effects would be observed, depending on the laser fluence that is applied.

Electrons that belong to structural defects in the lattice require a low energy to be detached from the crystal network. The detachment of electrons leads to the formation of an exciton, and the non-equilibrium carriers (electron–hole pairs) couple with the lattice system temporally and spatially, finally transferring most of their energy to the lattice. As a result, the system tries to recover equilibrium by distributing the excess of energy between carriers and lattices, leading to the segregation of the constituent elements such as Ag, W or O. The segregation of Ag from the constituent clusters is considered to be easier, due to the clusters containing Ag are located at the exterior places of the crystaline network^2^. Therefore, the clusters that should receive more energy from the incoming laser radiation and consecuentry being forced to leave the crystal network in a faster way are the Ag clusters, the clusters that are located at the surface of the crystals.

In consequence, the area subjected to the laser radiation where the departure of Ag takes place presents a large number of Ag vacancies, resulting in the transformation from an n-type to a p-type semiconductor. This could be attributed to the laser-induced formation of internal defects, enhancing the transfer and separation of photogenerated electron-hole pairs.

When the material undergoes a higher laser fluence, a large number of electrons are pulled out of the material’s surface, thus forming an electronic cloud. This effect causes the remaining ions to leave the surface of the materials by electromagnetic attraction or inelastic collisions^51,52^. The cloud formed by hot electrons, ions and some other detached species form a plasma known as plasma plume, the life-time of which is in the order of nanoseconds^53^. After the plasma plume is extinguished, the excess energy is released into the surrounding medium and the surface of the sample. Some of the elements forming the plasma are expelled into the surrounding medium and others are projected onto the surface of the sample. Ag, W and O elements experience an instantaneous nucleation leading to the formation of spherical NPs of Ag, and spherical NPs of a mixture of Ag, W and O, the shape taking on a spherical form due to this being the minimum energy shape for nanocrystals. Additionally, some elements do not have enough time to leave the α-Ag_2_WO_4_ network completely and form spheres attached to the surface of the sample. The lack of time may lead to a lack of energy, thereby promoting an effect that is similar to the one observed at low laser fluences.

When the sample undergoes the irradiation of an electron beam, the formation of Ag filaments takes 5 min at an energy of 10 kV. According to Longo *et al*., during the electron irradiation process, an electronic and structural disorder is introduced into the material through the clusters, which play a major role in the nucleation and growth of Ag filaments^2^. The electrons added to the material by the electron beam are transferred from one cluster to another through the crystal lattice, and Ag formation occurs by reducing clusters [AgO_2_] and [AgO_4_] in an orderly fashion and, to a lesser extent, through the cluster reduction of [WO_6_]. It is proposed that the segregation of Ag atoms by laser irradiation in femtoseconds occurs in a similar way, but in theory faster due to the fact that a femtosecond (10^−15^s) laser pulse is able to deliver peak power in the region of gigawatts in a shorter time than the conventional electronic relaxation time for most materials (10^−12^s). Therefore, due to the expected velocity of segregation, the morphology of Ag-segregated NPs tends to be different under electron beam radiation from that obtained when the sample is irradiated with femtosecond laser radiation.

The reasons for conducting this research are manifold and encompass the intellectual excitement of deriving macroscopic performance from molecular-level foundations. The extreme light-matter interaction of femtosecond pulses with α-Ag_2_WO_4_ has revealed itself as a method to produce silver tungstate nanocomposites in large areas of the sample, which is very interesting from the point of view of scaling up the process. Moreover, our investigation has demonstrated the laser light-driven synthesis of a rich variety of chemical species, which can be summarized as follows:

- Black species: When the sample is irradiated by the ultrafast laser following protocol I or II. In this case, (spherical) Ag nanoparticles and crystalline Ag clusters are formed on the surface of Ag_2-x_WO_4-δ_. We consider that this is a photochemical phenomenon and, therefore, it is not exclusively induced by ultrafast radiation. Moreover, we observed that the new α-Ag_2-x_WO_4-δ_ laser composition was stable against electron beam interaction, whereas the regions of the α-Ag_2_WO_4_ nanorods where Ag° was not generated led to Ag^0^ nanofilament growth during electron beam irradiation.
- Red cloud species: When the sample is irradiated by laser following protocol I (only at high fluences). This is a plasma-mediated ablation of the α-Ag_2_WO_4_ sample, yielding Ag_x_W_y_O_z_ nanoparticles. The nanoparticles have been produced due to a multiphoton absorption and the subsequent ionization process.

Furthermore, the bactericidal properties of the laser-irradiated samples increase dramatically. A 32-fold improvement can be observed compared to the non-laser irradiated material. We believe this work is instructive for the design of nanoparticles on a α-Ag_2_WO_4_ framework induced by femtosecond laser irradiation. Further efforts in this area are currently underway. In particular, work is being carried out on the tunability of the laser-induced effects by controlling and varying the laser power and the irradiation time, which may open up a new field in the synthesis of novel materials with a broad range of applications.

## Methods

**Figure 8:**
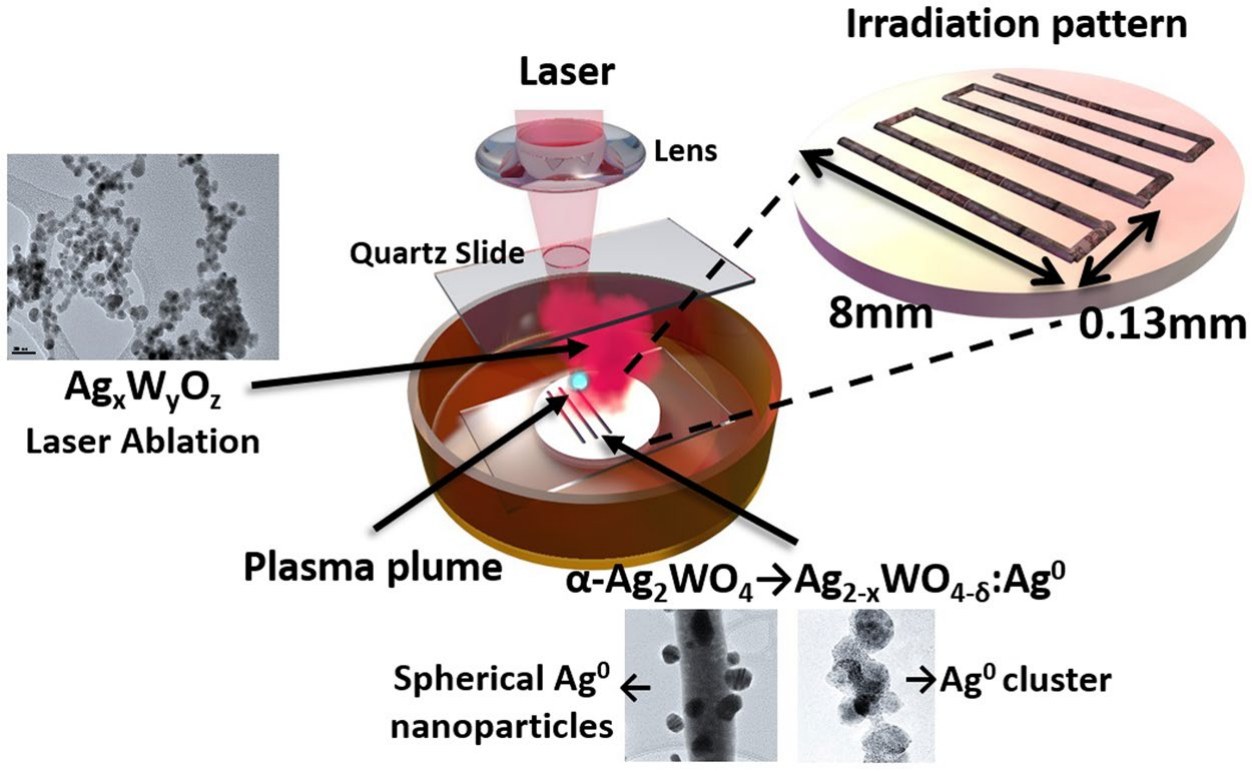
Schematic representation of the experimental procedure, representation of the observed results, and inset showing the representation of the irradiation pattern.

α-Ag_2_WO_4_ powders were prepared according to the co-precipitation procedure described by Longo *et al*.^17^. Powders were also pressed into pellets by a uni-axial press. α-Ag_2_WO_4_ pellets were irradiated with a Ti:sapphire laser (Femtopower Compact Pro, Femto Lasers) using 30 fs full width at half maximum (FWHM) pulses at the central wavelength of 800 nm, and a repetition rate of 1 kHz. To achieve more precise pulse compression at the sample, a programmable acousto-optic filter (DAZZLER, Faslite) was used. A laser beam with a diameter of 6 mm, at the 1/e^2^ point, and a mean power of 200 mW was focused onto the surface of a target pellet of α-Ag_2_WO_4_ with a spherical convex 75 mm lens in order to obtain a focal spot with a diameter of 20.6 µm and in this way achieve a laser fluence of 60 J/cm^2^, which is a considerably high fluence. The α-Ag_2_WO_4_ sample was placed at the bottom of a quartz cuvette attached to a two-dimensional motion-controlled stage moving at a constant speed of 0.45 mm/s in the focus plane perpendicular to the laser beam in a stair-like pattern. A scheme of the experimental procedure is shown in Fig. 8, where the results obtained are also presented pictorially, and an irradiation stair-like pattern is depicted in the inset. A complete video of laser-matter interaction can be found in the Supplementary Material.

Additional experiments were conducted to better understand the current behavior.

1. In order to spread the energy through a larger irradiation spot in the sample in order to avoid the creation of a plasma plume, the position of the α-Ag_2_WO_4_ was set 8 mm closer to the convex lens so as to obtain an irradiation spot with a diameter of 84.3 µm, the incoming power was set to 200 mW, and therefore the irradiation laser fluence was 3.6 J/cm^2^. The highest fluence, obtained at an irradiation spot with a diameter of 20.6 µm, was accompanied by the production of a plasma plume, visible to the naked eye, and the ablation of material. However, when the irradiation spot was set to 84.3 µm the plasma was no longer observed but there was still an explicit interaction between the laser and the α-Ag_2_WO_4_ as it changed its color to black without material ablation.
2. α-Ag_2_WO_4_ samples in powder state and deposited in a large vessel were irradiated with 200 mW, matching the focal plane of the lens with the surface of the powder, to obtain an irradiation spot of 20.6 µm, and the vessel was attached to the stage moving at the same constant velocity along the same stair-like pattern. Under these experimental conditions, the black color was still formed over the powder but the species from the ablation plume, light red in color, were collected in the vessel as can be observed in Fig. 1D. To collect the species in the red cloud, a microscope coverslip was located in the path of the laser beam so that the cloud species in the plasma were naturally attached to the coverslip.

To determine the crystallinity of α-Ag_2_WO_4_ samples before any treatment, a D/Max-2500PC (Rigaku, Japan) diffractometer with Cu Kα 108 radiation (λ = 0.154 nm) was used in a 2θ range from 10° to 70° at a scan velocity of 2°/min with an step of 0.02°. The XRD patterns of the samples indicate an orthorhombic structure with a space group of Pn2n, in agreement with the JCPDS database (PDF34-0061). The lattice parameters were a = 1.082 nm, b = 1.201 nm, and c = 0.59 nm. All the peaks were sharp in nature, indicating a high degree of crystallinity.

To compare the ultrafast laser-irradiated samples with TEM-treated samples, a non-laser irradiated α-Ag_2_WO_4_ sample was treated by an electron beam using a field-emission scanning electron microscope (model Inspect F50, FEI Company, Hillsboro, OR) at 10 kV for 5, 10, 15 and 20 minutes (see the evolution of the growth of Ag NPs at different times in Figure S1 in the Supplementary Material).

To characterize the structural changes that occurred in the irradiated regions, the laser-irradiated samples were characterized by HR-TEM. This was performed using a Jem-2100 LaB6 (Jeol) HR-TEM with an accelerating voltage of 200 kV coupled with an INCA Energy TEM 200 (Oxford) energy dispersive X-ray spectrometer. The same instrument was used to perform TEM, electron diffraction, and microanalysis measurements to characterize structural changes. Powder samples were prepared by depositing a representative sample of the powder directly onto holed carbon-coated Cu grids, whereas the pellets were prepared by ultramicrotomy. The pellets were embedded in an epoxy resin and sliced at room temperature in an RMC Utracut (Powertome XL model).

And finally, to test the bactericidal activity of the samples, planktonic cultures of methicillin-resistant Staphylococcus aureus (ATCC 33591) strains were grown in mannitol salt agar (MSA, Acumedia Manufactures Inc. Baltimore, Maryland, USA) at 37 °C for 48 hrs. The cultures were then diluted and developed in 10 mL of TSB (Acumedia Manufactures, Inc. Baltimore, Maryland, USA) media to an optical density corresponding to, approximately, 10^5^ CFU ml^−1^.

After the exposure of MRSA to non-treated and laser-treated α-Ag_2_WO_4_ powders, a confocal laser scanning microscope (CLSM - Zeiss LSM 780) was used to take fluorescence images of the samples in order to determine the concentration of still-living bacteria, and the morphology and population was analyzed by SEM (SEM-FEG - JEOL 7500 F). For CLSM, MRSA specimens were marked with the BacLight (Invitrogen) viability kit, in accordance with the manufacturer’s recommendations.

## Electronic supplementary material

Supplementary Material

Supplementary Movie
