# Supplementary material for: Towards the scale-up of the formation of nanoparticles on α-Ag2WO4 with bactericidal properties by femtosecond laser irradiation
Source: Sci Rep. 2018 Jan 30;8:1884. doi: 10.1038/s41598-018-19270-9 (PMC5789880; doi:10.1038/s41598-018-19270-9)
Supplement: Supplementary file 1 — Supplementary Material [file 41598_2018_19270_MOESM1_ESM.pdf]

Supplementary Material for:

Towards the scale-up of the formation of nanoparticles on α-Ag_2WO_4 with bactericidal properties by femtosecond laser irradiation

Marcelo Assis[1], Eloisa Cordoncillo[2], Rafael Torres-Mendieta[3], Héctor Beltrán-Mir[2], Gladys Mínguez-Vega[4], Regiane Oliveira[1], Edson R. Leite[1], Camila C. Foggi[5], Carlos E. Vergani[5], Elson Longo[1,*], and Juan Andrés[6,*]

[1]CDMF-UFSCar, Universidade Federal de São Carlos, P.O. Box 676, CEP, 13565-905 São Carlos-SP, Brazil.

[2]Department of Inorganic and Organic Chemistry, University Jaume I (UJI), Castelló 12071, Spain.

[3]Institute for Nanomaterials, Advanced Technologies and Innovation, Technical University of Liberec, Studentská 1402/2, 461 17 Liberec, Czech Republic.

[4]GROC·UJI, Institut de Noves Tecnologies de la Imatge (INIT, University Jaume I (UJI), Castelló 12071, Spain.

[5]FOAr-UNESP, Universidade Estadual Paulista, P.O. Box 1680, 14801903 Araraquara, SP Brazil.

[6]Department of Analytical and Physical Chemistry, University Jaume I (UJI), Castelló 12071, Spain.

*elson.liec@gmail.com

*andres@qfa.uji.es

1. Supplementary image of the evolution of Ag filaments created by electron beam, and X ray spectra.

2. Video: Experimental set up.

1. Supplementary image of the evolution of Ag filaments created by electron beam, and X ray spectra.

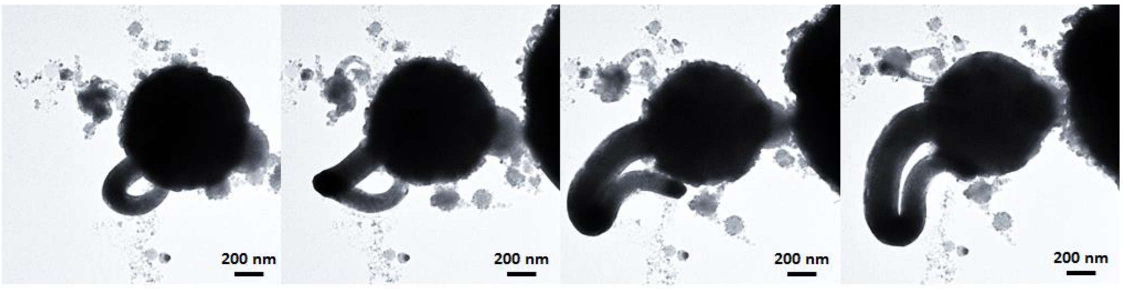

Figure S1. Evolution with time of the Ag filament growth in α-Ag_2WO_4 during the exposition to the electron beam. After, A) 5, B) 10, C) 15 and D) 20 minutes.

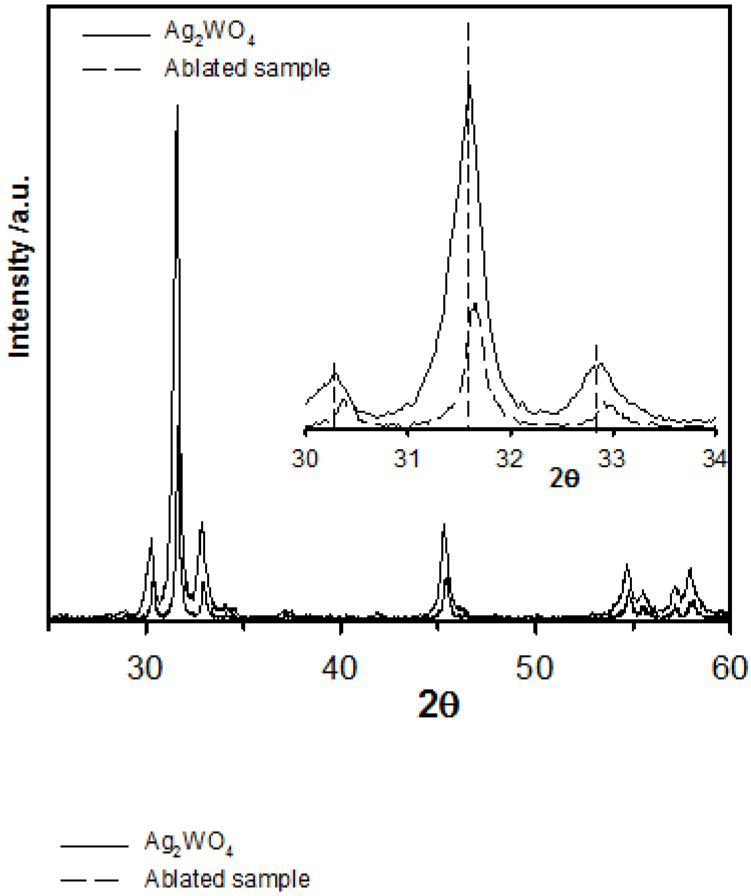

Figure S2. X-ray diffraction of α-Ag_2WO_4 and α-Ag_2WO_4 laser irradiated in femtoseconds and evidencing the peak displacement of the irradiated sample (inset).

2. Video: Experimental set up

α-Ag_2WO_4 pellet under laser irradiation in a 8 X 8 mm area in a stair-like pattern, and a representation of the chemical species obtained after the laser treatment.
